# Cholera toxin B subunit induces local curvature on lipid bilayers

**DOI:** 10.1002/2211-5463.12321

**Published:** 2017-10-10

**Authors:** Weria Pezeshkian, Lina J. Nåbo, John H. Ipsen

**Affiliations:** ^1^ Center for Biomembrane Physics (MEMPHYS) Department of Physics, Chemistry and Pharmacy (FKF) University of Southern Denmark Odense Odense M Denmark

**Keywords:** endocytosis, ganglioside, peripheral proteins

## Abstract

The B subunit of the bacterial cholera toxin (CTxB) is responsible for the toxin binding to the cell membrane and its intracellular trafficking. CTxB binds to the monosialotetrahexosyl ganglioside at the plasma membrane of the target cell and mediates toxin internalization by endocytosis. CTxB induces a local membrane curvature that is essential for its clathrin‐independent uptake. Using all‐atom molecular dynamics, we show that CTxB induces local curvature, with the radius of curvature around 36 nm. The main feature of the CTxB molecular structure that causes membrane bending is the protruding alpha helices in the middle of the protein. Our study points to a generic protein design principle for generating local membrane curvature through specific binding to their lipid anchors.

**Abbreviations**
aaMD: all‐atom molecular dynamics
CTxB: cholera toxin B subunit
DOPC: 1,2‐dioleoyl‐sn‐glycero‐3‐phosphocholine
GM1: monosialotetrahexosyl ganglioside
S‐GM1/CTxB: system containing a CTxB and a bilayer mixture of DOPC and S‐GM1
S‐GM1: GM1 with a saturated acyl chain
U‐GM1/CTxB: system containing a CTxB and a bilayer mixture of DOPC and U‐GM1
U‐GM1: GM1 with an unsaturated acyl chain

The bacterial cholera toxin is an example of a pathogenic toxin that uses endocytosis for entry into host cells. It is secreted by the Gram‐negative bacterium *Vibrio cholerae*, which is associated with severe diarrhea and responsible for the death of ~ 10^5^ people annually 1. The protein is a member of the AB5 toxin families that are comprised of a single catalytic A subunit and a membrane binding B subunit (CTxB). CTxB binds to monosialotetrahexosyl ganglioside (GM1) of the plasma membrane of the host cell, which subsequently drives its internalization into the cell 2. CTxB is a homopentameric protein with radius of 3.6 nm, which is similar to the B subunit of Shiga toxin (STxB) 3 (Fig. 1A), while the thickness of the protein (4.1 nm) is larger than STxB (Fig. 1B). Both CTxB and STxB internalize through clathrin‐dependent 4, 5 and clathrin‐independent endocytosis 6, 7, 8, 9, 10. Characteristic for the clathrin‐independent endocytosis is the formation of narrow tubular invaginations that are induced by toxic particles binding to the membrane. The formation of tubular invaginations requires a small increment of local curvature generated by toxic proteins 3 and clustering of these particles in a domain 11. The small spontaneous curvature of the domain leads to a spontaneous tension 12 that subsequently drives the membrane tubular invagination. Therefore, a small local protein‐induced membrane curvature and clustering of proteins are enough for the formation of tubular invaginations 12. Hence, it is of interest to understand how the toxic proteins induce local curvature upon binding and how much spontaneous curvature they generate. Despite many simulation studies of cholera toxin and its B subunit, neither a quantitative estimation of nor a principle behind CTxB‐induced membrane curvature has been reported 13, 14, 15, 16. Therefore, we have performed all‐atom molecular dynamics (aaMD) simulations to characterize, at the atomistic length scale, the effect of binding of a single CTxB pentamer on the lipid bilayer curvature.

**Figure 1:**
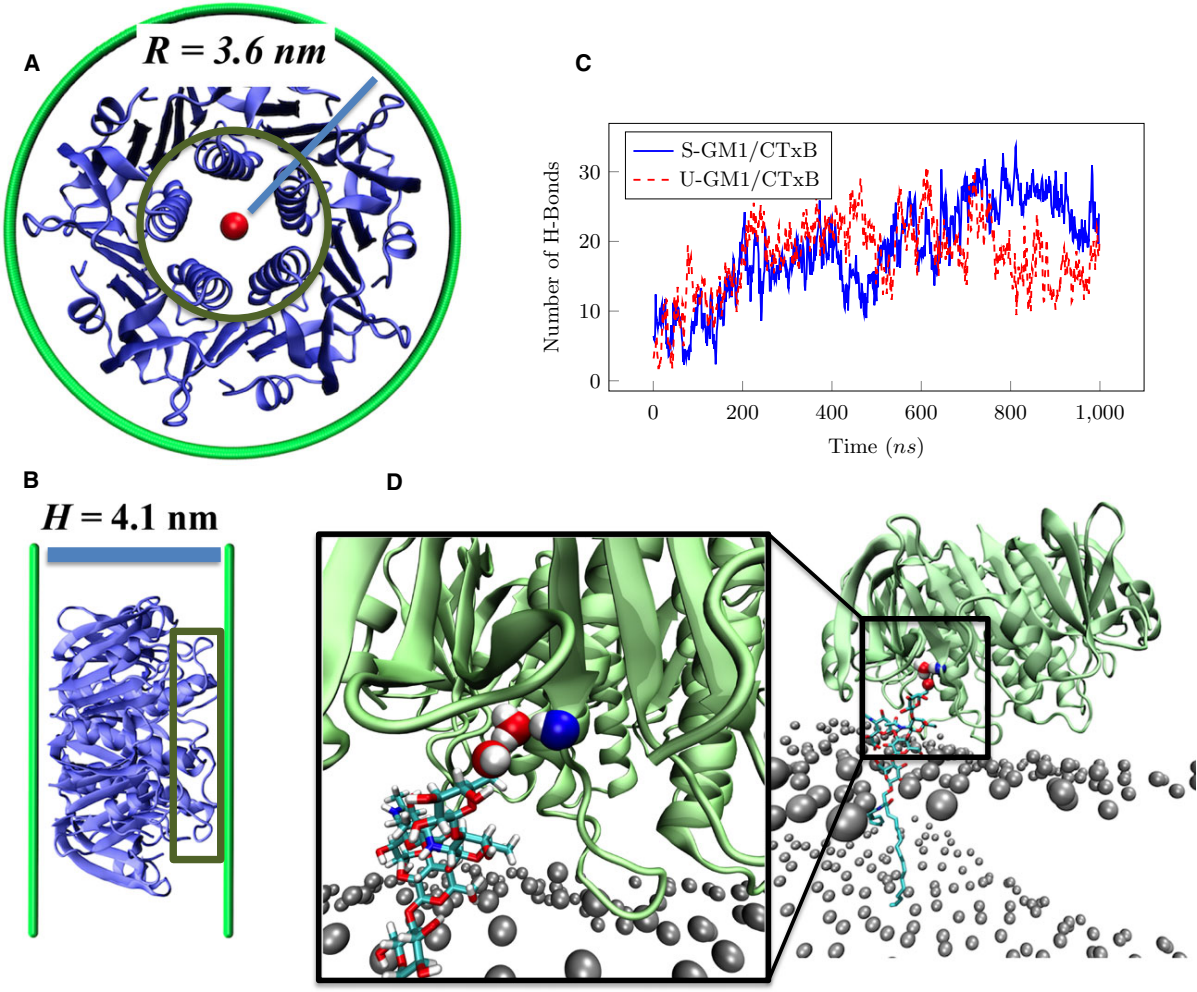
CTxB size and binding to a lipid bilayer containing GM1 lipids. (A) Smallest circle that wraps around CTxB. Inner green circle shows the alpha helices, which are located in the middle of the protein. (B) Lateral view of CTxB. Green rectangular: Alpha helices are protruding away from the binding face of the CTxB with respect to the protein edges, (C) number of the direct hydrogen bonds between CTxB and GM1 lipids as a function of time, (D) a representation of a water‐mediated hydrogen bond between CTxB and one bound GM1. The gray beads are the phosphor atoms of the DOPC lipids (DOPC lipids and other bound GM1 are not shown for clarity).

## Materials and methods

**Figure 2:**
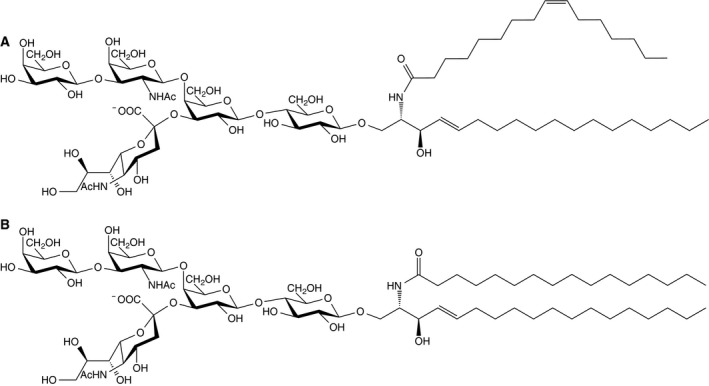
Two different types of ganglioside GM1: (A) GM1 with U‐GM1 and (B) GM1 with S‐GM1.

Each simulated system consists of a single CTxB pentamer and a symmetric lipid bilayer containing 414 1,2‐dioleoyl‐sn‐glycero‐3‐phosphocholine (DOPC) and 36 saturated or unsaturated GM1 lipids (Fig. 2). We will refer to the system containing saturated (unsaturated) GM1 as S‐GM1/CTxB (U‐GM1/CTxB). Each system was simulated for 1 μs to allow CTxB to bind GM1 lipids. Then, from the last 100‐ns trajectory of each simulation, two different snapshots were chosen as starting configurations for two replicas that subsequently were simulated for 500 ns with new different initial random velocities. The simulations were performed using gromacs 17, 18 and the CHARMM36 force field 19, 20 with the TIP3P water model 21. The CTxB crystal structure was obtained from ref 22. Both CTxB and GM1 are charged molecules (CTxB has 10 positive charges and GM1 has one negative charge); therefore, 26 K^+^ ions were added to neutralize the system. Electrostatic interactions were treated with particle‐mesh Ewald (PME) with a short‐range cutoff at 1.2 nm, and van der Waals interactions were switched off between 1.0 and 1.2 nm. The system temperature and pressure were kept constant at 37 °C and 1 bar using Nose–Hoover temperature coupling 23, 24 and semi‐isotropic Parrinello–Rahman barostat 25 after equilibration for 50 ns using the Berendsen pressure and temperature coupling 26. Bonds containing hydrogen atoms were constrained using the LINCS algorithm 27. Finally, the leap frog integrator was used with a time step of 2 fs.

## Results and Discussion

**Figure 3:**
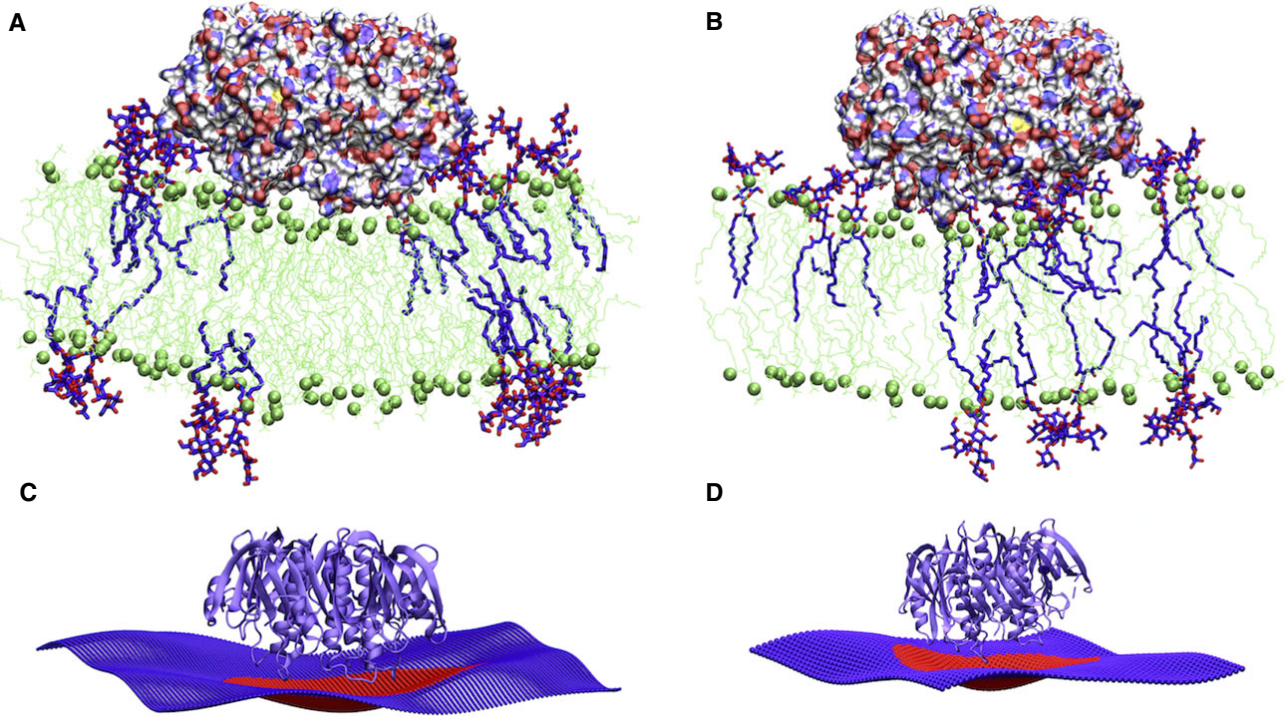
Snapshot of CTxB inducing local curvature. (A) A cross section image of the U‐GM1/CTxB system showing that CTxB induces local curvature upon binding to the membrane. Green: DOPC lipids; purple: GM1 lipids. (B) A cross section image of the S‐GM1/CTxB system and (C) reconstruction of the bilayer surface in (A) by fitting to Eqn (1). Red indicates the area under the protein. (D) Reconstruction of the bilayer surface in (B).

After 1 μs simulation time, CTxB is effectively bound to the bilayer through both direct hydrogen bonding to GM1 (Fig. 1C), as it is predicted by aaMD simulations 13 and protein crystallography 22, and water‐mediated hydrogen binding (Fig. 1D and Movie S1) as suggested from the crystal structure 22. Figure 3 shows snapshots of a CTxB binding to a membrane containing GM1 lipids inducing an apparent membrane curvature as previously reported 13, 14. However, the snapshots of a curving membrane obtained in an aaMD simulation do not provide numerical or theoretical evidence for the local membrane curving ability of CTxB. It may simply signify curvature fluctuations, which is present even in the absence of any proteins attached to the membrane. The quantification of the local membrane curvature associated with a bound or imbedded protein involves the time average of an appropriate membrane curvature quantifier 3.

To quantify CTxB‐induced curvature, we first obtain the position of the bilayer surface numerically from snapshots during the course of the simulation. From this surface, the local mean bilayer curvature can be found as $C_{0}\left(x,y\right)=-\frac{1}{2}▿\hat{n}$, where $\hat{n}$ is a unit vector that is normal to the bilayer surface. The position of the bilayer surface (*z*_mem_ (*x,y*) in Monge representation) is defined as the arithmetic mean of its monolayers’ interface positions (*z*_up_ (*x,y*), *z*_low_ (*x,y*)). They are found by fitting the phosphorus atom coordinates of the DOPC lipids in each monolayer to a Fourier series in two dimensions as

(1) $$z\left(x,y\right)=\sum_{n=-N_{x}}^{N_{x}}\sum_{m=-N_{y}}^{N_{y}}A_{nm}\left(\text{cos}\left[p_{n}x+q_{m}y\right]+\text{sin}\left[p_{n}x+q_{m}y\right]\right),$$

where $p_{n}=\frac{2\pi n}{L_{x}},q_{m}=\frac{2\pi m}{L_{y}}$ and *L*_*x*_ and *L*_*y*_ are the bilayer extensions in *x* and *y* directions, respectively. Here, we have used *N*_*x*_ = *N*_*y*_ **= 2. Note that in this formulation, an inward (upward) bending will be represented by a positive (negative) curvature.

**Figure 4:**
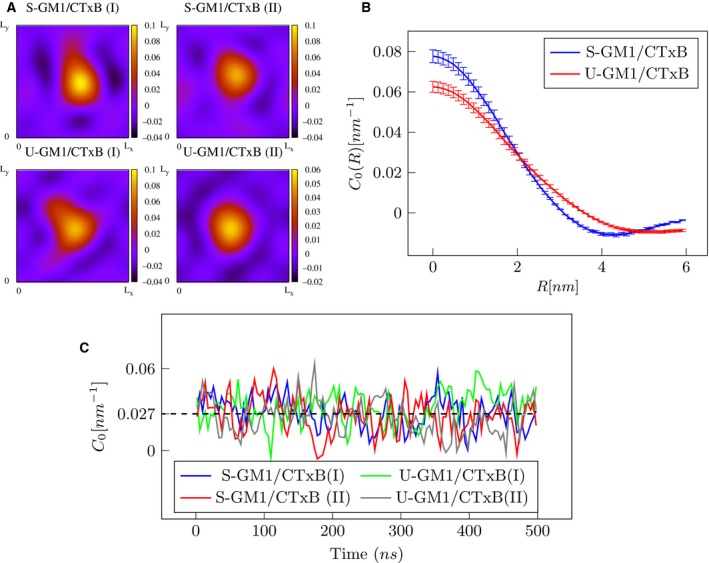
CTxB induces local curvature on lipid bilayers. (A) 2D map of the average curvature on the membrane. Top row; S‐GM1/CTxB systems. Bottom row; U‐GM1/CTxB systems. The roman numbers are used to label different replicas for each system. (B) Radial curvature profile obtained from the center of CTxB using the maximum‐likelihood principle to generate the average between the replicas (C) Local curvature of a membrane segment under the protein as a function of time for different systems. The dashed line shows the average value of the curvature.

To perform time averaging of the local bilayer curvature, we have refined the trajectories of the simulations so that the CTxB is always in the center of the simulation box. Figure 4A shows 2D maps of the average curvature (average over 500 ns) on the surface of the bilayer for different systems. It is obvious that the most curved area is at the center of the simulation box, where the protein is located, which indicates that the CTxB is responsible for membrane bending. For more clarification, we calculated the radial profile of the membrane curvature as $C_{0}\left(R\right)=\frac{1}{2\pi }\int_{0}^{2\pi }C_{0}\left(R,\varphi \right)d\varphi$, where *R* and φ are radial distance (from the center of the protein in the plane of the bilayer) and azimuth angle in cylindrical coordinates, respectively. As it is shown in Fig. 4B, *C*_0_(*R*) decreases when *R* increases and it vanishes at around 3 nm, approximately the distance where GM1 lipids are bound to the protein.

The total curvature for a membrane with a periodic boundary condition is zero. To quantify the curvature induced by CTxB, we therefore calculated the local curvature of a bilayer segment under the protein as $C_{0}=\frac{1}{A_{p}}\int_{A_{p}}C\left(x,y\right)dxdy$, where *A*_p_ is the projected area of CTxB on the membrane plane. Figure 4C shows the local curvature as a function of time for different systems. The time‐averaged values of the local curvature are 0.032 ± 0.003 and 0.024 ± 0.003 nm^−1^ for different replicas of the U‐GM1/CTxB system and 0.028 ± 0.002 and 0.025 ± 0.002 nm^−1^ for S‐GM1/CTxB replicas. Using the maximum‐likelihood principle, the average values are 0.028 ± 0.002 nm^−1^ and 0.027 ± 0.001 for U‐GM1/CTxB and S‐GM1/CTxB systems, respectively. These results suggest that CTxB induces local curvature, with the radius of curvature (inverse of mean curvature) around 36 nm, which is not influenced by GM1 lipid chain saturation level. This level of curvature is sufficient for the generation of tubular membrane invaginations 3.

We previously described that bending of the membrane is induced by binding of STxB to its lipid anchors in different orientations and heights with respect to the membrane surface. Most importantly, Gb3 lipid head groups that are bound to the binding site 3 (at the middle of the binding face of STxB) are normal to the protein surface, which leads to a lower height of the hydrophobic solvent interface compared to the lipids bound to the sites at the edges of STxB 3. However, CTxB has only one type of binding site (one per monomer), and therefore, the bound GM1 lipids are all similar in orientation and height. Interestingly, CTxB alpha helices, which are located in the middle of the protein, are protruding away from the binding face of the CTxB with respect to the protein edges (Fig. 1A–B). All CTxB binding sites are located around its edges, so by binding to the membrane, the alpha helices are placing down the lipids underneath compared to the lipids close to the binding sites. This drives the membrane to bend upon binding of the CTxB proteins. In other words, a bound CTxB applies two types of vertical forces to the bilayer: an upward force around its edges and a downward force through its alpha helices at the protein center resulting in membrane bending. We therefore suggest that the lengthy alpha helices at the center of the CTxB ring play the role of STxB binding site 3 for generating local curvature. Despite different molecular design of STxB and CTxB, the resulting physical structure induces a local curvature based on a similar principle. Based on these results, we hypothesize that the combination of the positioning of specific binding units and protein shape provides a new structural motif for curvature generation in membranes, which can be utilized by pathogens such as bacterium *V. cholerae*. The induced local curvature is the result of the interfacial structure of the CTxB–GM1 complex, involving many hydrogen bonds (Fig. 1C). Therefore, we expect that lipid bilayer composition plays the secondary role. However, the lipid composition may be important for CTxB clustering and subsequently tubular invagination; for example, the presence of cholesterol‐induced raft domains enriched with GM1 increases the efficiency. According to the model we described here, a reduced number of the bound GM1 lipids results in a lower induced local curvature. This is in line with the observation of diminished uptake efficiency of CTxB variants with fewer binding sites; for example, the smaller local mean curvature results in lower partitioning into high curvature transport vesicle structures such as sorting endosomes 28.

**Figure 5:**
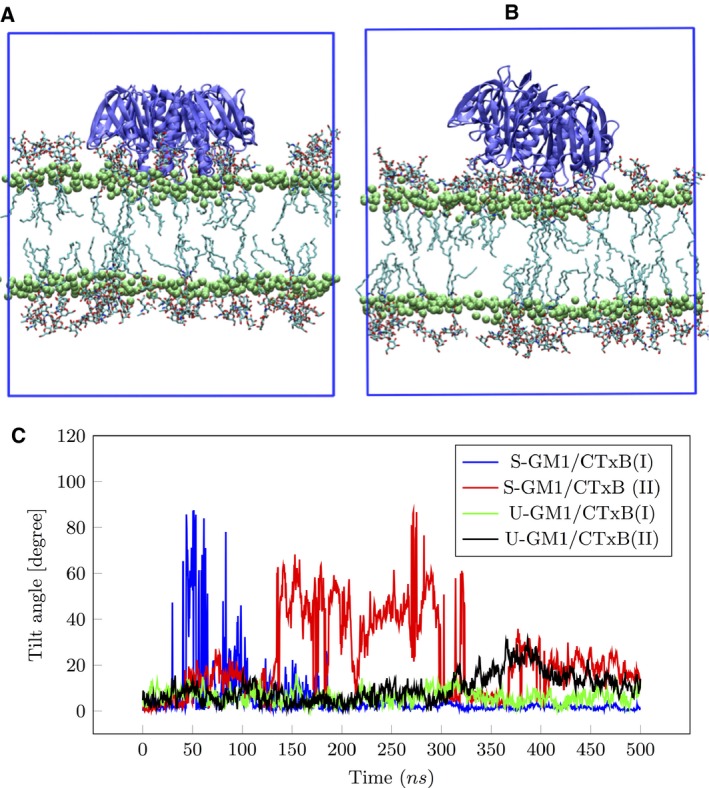
CTxB orientation on a lipid bilayer. (A) and (B) Nontilted configuration and tilted configurations, respectively. The green beads are the phosphor atom of the DOPC lipids. For clarity, only the GM1 lipids are shown. (C) Angle between protein normal and bilayer normal as a function of time for different systems. U‐GM1/CTxB is mostly found in nontilted configuration.

Previously, it has been observed that CTxB binds to a bilayer in a tilted orientation 13, 14. To test this, we determined three principal axes of CTxB and have chosen the axis with the largest moment of inertia as the protein normal orientation. Our results suggest that CTxB binds to the bilayer in both tilted and nontilted configurations (Fig. 5A,B). In the nontilted configuration, the protein normal vector is nearly perpendicular to the bilayer surface and only has some thermally induced fluctuations, while in the tilted configuration, the protein normal vector has a nonperpendicular orientation and at least one of its monomers is not bound to any GM1 lipids. Figure 5C shows the angle between the protein normal and the bilayer normal as a function of time for different systems. The graph shows that for the S‐GM1/CTxB systems, the protein can go from both a tilted to nontilted (blue curve) and a nontilted to tilted (red curve) configuration. However, for U‐GM1/CTxB systems, the tilted configuration was observed only for a short period of time (gray curve). The orientation of the bound CTxB on the membrane depends on which hydrogen bonds are saturated. In general, the orientation of the binding residues of the CTxB and head groups of GM1 does not support that all the possible hydrogen bonds become saturated especially when they are constrained to a lipid bilayer surface. Therefore, the large binding degeneracy and energetically close binding configurations can give rise to large varieties of CTxB orientations.

## Author contributions

JHI conceived the study. WP performed the simulations. LJN provided structure and FF parameter for GM1. All authors discussed the results and commented on the manuscript at all stages.

## Supporting information


**Movie S1**. Showing a water‐mediated hydrogen bond between a single CTxB and GM1 lipid.
